# Correction: Neurological and Histological Consequences Induced by *In Vivo* Cerebral Oxidative Stress: Evidence for Beneficial Effects of SRT1720, a Sirtuin 1 Activator, and Sirtuin 1-Mediated Neuroprotective Effects of Poly(ADP-ribose) Polymerase Inhibition

**DOI:** 10.1371/journal.pone.0106166

**Published:** 2014-08-13

**Authors:** The PLOS ONE Staff

## Notice of Republication

This article was republished on July 30, 2014, to correct the placement of the figures which were mistakenly published as Supporting Information. Please download this article again to view the correct version. The originally published, uncorrected article and the republished, corrected article are provided here for reference.

## Supporting Information

File S1 Originally published, uncorrected article.
(PDF)

File S2 Republished, corrected article.
(PDF)
