# Supplementary figures and images for: Neurological and Histological Consequences Induced by In Vivo Cerebral Oxidative Stress: Evidence for Beneficial Effects of SRT1720, a Sirtuin 1 Activator, and Sirtuin 1-Mediated Neuroprotective Effects of Poly(ADP-ribose) Polymerase Inhibition
Source: PLoS One. 2014 Feb 21;9(2):e87367. doi: 10.1371/journal.pone.0087367 (PMC3931616; doi:10.1371/journal.pone.0087367)

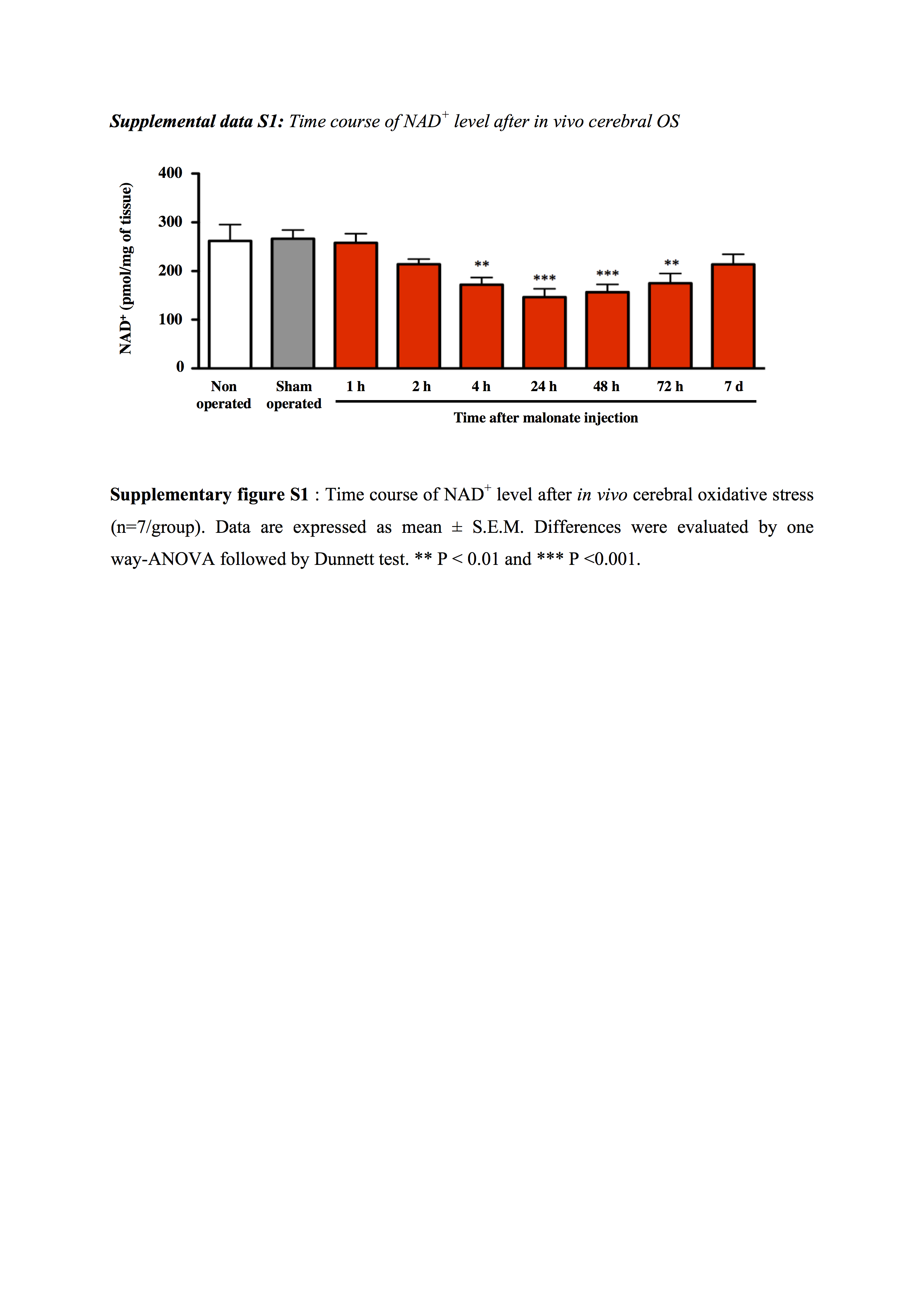

Supplement: Data S1 — Time course of NAD+ level after in vivo cerebral oxidative stress (n = 7/group). Data are expressed as mean ± S.E.M. Differences were evaluated by one way-ANOVA followed by Dunnett test. **P<0.01 and ***P<0.001. (TIFF) [file pone.0087367.s001.tif]
