# Supplementary material for: Correction: Neurological and Histological Consequences Induced by In Vivo Cerebral Oxidative Stress: Evidence for Beneficial Effects of SRT1720, a Sirtuin 1 Activator, and Sirtuin 1-Mediated Neuroprotective Effects of Poly(ADP-ribose) Polymerase Inhibition
Source: PLoS One. 2014 Aug 13;9(8):e106166. doi: 10.1371/journal.pone.0106166 (PMC4132092; doi:10.1371/journal.pone.0106166)
Supplement: File S1 — Originally published, uncorrected article. (PDF) [file pone.0106166.s001.pdf]

PLOS ONE

Neurological and Histological Consequences Induced by *In Vivo* Cerebral Oxidative Stress: Evidence for Beneficial Effects of SRT1720, a Sirtuin 1 Activator, and Sirtuin 1-Mediated Neuroprotective Effects of Poly(ADP-ribose) Polymerase Inhibition

Cindy Gueguen, Bruno Palmier, Michel Plotkine, Catherine Marchand-Leroux, Valérie C. Bessson*

Université Paris Descartes, Pharmacologie de la Circulation Cérébrale - EA4475, Faculté des Sciences Pharmaceutiques et Biologiques, Paris, France

Abstract

Poly(ADP-ribose)polymerase and sirtuin 1 are both NAD^+-dependent enzymes. *In vitro* oxidative stress activates poly(ADP-ribose)polymerase, decreases NAD^+ level, sirtuin 1 activity and finally leads to cell death. Poly(ADP-ribose)polymerase hyperactivation contributes to cell death. In addition, poly(ADP-ribose)polymerase inhibition restores NAD^+ level and sirtuin 1 activity *in vitro*. *In vitro* sirtuin 1 induction protects neurons from cell loss induced by oxidative stress. In this context, the role of sirtuin 1 and its involvement in beneficial effects of poly(ADP-ribose)polymerase inhibition were evaluated *in vivo* in a model of cerebral oxidative stress induced by intrastriatal infusion of malonate in rat. Malonate promoted a NAD^+ decrease that was not prevented by 3-aminobenzamide, a poly(ADP-ribose)polymerase inhibitor, at 4 and 24 hours. However, 3-aminobenzamide increased nuclear SIRT1 activity/expression ratio after oxidative stress. Malonate induced a neurological deficit associated with a striatal lesion. Both were reduced by 3-aminobenzamide and SRT1720, a sirtuin 1 activator, showing beneficial effects of poly(ADP-ribose)polymerase inhibition and sirtuin 1 activation on oxidative stress consequences. EX527, a sirtuin 1 inhibitor, given alone, modified neither the score nor the lesion, suggesting that endogenous sirtuin 1 was not activated during cerebral oxidative stress. However, its association with 3-aminobenzamide suppressed the neurological improvement and the lesion reduction induced by 3-aminobenzamide. The association of 3-aminobenzamide with SRT1720, the sirtuin 1 activator, did not lead to a better protection than 3-aminobenzamide alone. The present data represent the first demonstration that the sirtuin 1 activator SRT1720 is neuroprotective during *in vivo* cerebral oxidative stress. Furthermore sirtuin 1 activation is involved in the beneficial effects of poly(ADP-ribose)polymerase inhibition after *in vivo* cerebral oxidative stress.

Citation: Gueguen C, Palmier B, Plotkine M, Marchand-Leroux C, Besson VC (2014) Neurological and Histological Consequences Induced by *In Vivo* Cerebral Oxidative Stress: Evidence for Beneficial Effects of SRT1720, a Sirtuin 1 Activator, and Sirtuin 1-Mediated Neuroprotective Effects of Poly(ADP-ribose) Polymerase Inhibition. PLoS ONE 9(2): e87367. doi:10.1371/journal.pone.0087367

Editor: Jaya Padmanabhan, University of S. Florida College of Medicine, United States of America

Received July 9, 2013; **Accepted** December 20, 2013; **Published** February 21, 2014

Copyright: © 2014 Gueguen et al. This is an open-access article distributed under the terms of the Creative Commons Attribution License, which permits unrestricted use, distribution, and reproduction in any medium, provided the original author and source are credited.

Funding: This work was supported partly by Université Paris Descartes and by a grant from the Fondation des Gueules Cassées. The funders had no role in study design, data collection and analysis, decision to publish, or preparation of the manuscript.

Competing Interests: The authors have declared that no competing interests exist.

* E-mail: valerie.besson@parisdescartes.fr

Introduction

Oxidative stress (OS) is involved in physiopathology of acute cerebrovascular diseases such as stroke and traumatic brain injury [1,2]. It results from an unbalance between prooxidant and antioxidant systems. Brain is particularly vulnerable to OS as it is an important consumer of oxygen and it possesses low levels of antioxidants compared to other organs. Excessive production of reactive oxygen species causes lipid peroxidation, protein and DNA oxidation, leading to cell death [3]. Particularly, one response to DNA damage is poly(ADP-ribose)polymerase (PARP) activation.

PARP is a NAD^+-dependent nuclear enzyme that participates to DNA repair. Its excessive activation during OS contributes to energetic depletion and consequently to cell death [2,4,5]. It was widely demonstrated that PARP inhibition is beneficial in both *in vitro* and *in vivo* OS situations [6]. Indeed PARP inhibition decreased hydrogen peroxide-induced macrophage cell death [7] and was neuroprotective after *in vivo* cerebral OS [8]. Furthermore, *in vitro* data showed that PARP activation limited other NAD^+-dependent enzymes activity during OS, in particular sirtuin 1 (SIRT1), probably due to substrate depletion [9].

SIRT1 is a NAD-dependent deacetylase that have numerous targets that confers it a lot of biological functions. SIRT1 plays a significant role in histone post-translational modifications and chromatin-related functions, in the regulation of p53 expression and function, and in DNA damage response [10]. SIRT1 induction prevented *in vitro* OS-induced cell death [11]. Furthermore, its inhibition during wallerian degeneration exacerbated neuronal death whereas its activation protects neurons from cell loss [12]. These data suggested a beneficial role of SIRT1 activation on cell survival.

In vitro studies showed that OS activates PARP, decreases NAD^+ level, SIRT1 activity and finally leads to cell death [9,13–15]. *In*

vitro PARP inhibition restored NAD pool and SIRT1 activity in cells after hydrogen peroxide treatment [9,14,15] and prevented hydrogen peroxide-induced astrocyte cell death [16]. Conventionally, beneficial effects of PARP inhibition were explained as a consequence of NAD^+ preservation, thus it may allow NAD^+ use for SIRT1 activity. To our knowledge, there was no information dealing with the role of SIRT1 and its involvement in beneficial effects of PARP inhibition in the consequences of an *in vivo* cerebral OS. In this study, we used a rodent model of cerebral OS induced by malonate administration which causes oxygen and nitrogen reactive species production [8,17,18] and PARP activation [8]. In the first part, we evaluated the role of PARP on NAD^+ depletion, SIRT1 expression and activity. In the second part, we studied the role of SIRT1 and its implication in beneficial effects of PARP inhibition on neurological and histological OS consequences using treatment with a PARP inhibitor, a SIRT1 activator, a SIRT1 inhibitor, alone or in combination.

Materials and Methods

Animals

Male Sprague-Dawley rats (300–330 g) were supplied by Elevage Janvier laboratories (Le Genest-Saint-Isle, France). Animals were housed under standard conditions (temperature- and light-controlled) with free access to food and water. Animal care and all the experiments were performed in compliance with the ethical approvals stipulated by the Animal Ethics Committee of Paris Descartes University (registered number: CEEA34.CML.025.11), with the French regulations and the European Communities Council Directive of September 20, 2010, 2010/63/UE, on the protection of animals for experimental use and conformed to the *Guide for the Care and Use of Laboratory Animals* published by the U.S. National Institutes of Health (publication 85–23, revised 1996). All surgery was performed under chloral hydrate (or pentobarbital for brain remove) anaesthesia, and all efforts were made to minimize suffering.

Induction of *in vivo* Cerebral Oxidative Stress

Rats were anaesthetized with chloral hydrate (350 mg/kg, i.p.) and placed on a stereotaxic frame. During surgery, animals were positioned on a heating blanket (Harvard) to maintain body normothermia (37.5±0.5°C). A 3 M solution of malonate (M4795, Sigma) was prepared in 0.9% NaCl and pH was adjusted to 7.4. The scalp was incised and a craniotomy was performed at the following coordinates: 3.5 mm lateral to the bregma, 0 mm posterior to the bregma [19] [20]. A cannula (Sofijet 30 G) was placed at a depth of 7 mm below the surface of the skull and left in place for 5 minutes. One microliter of 3 M malonate solution (corresponding to 3 μmoles) was infused in the left striatum during 5 minutes *via* the cannula using a pump (Infors AG HT type Precidor). The injection cannula was left in place for a further 5 minutes to minimize the risk of retrograde leakage of the injected solution. Sham-operated rats underwent the same surgery and were given an intrastriatal injection of the same volume of 0.9% NaCl. The scalp was sutured and the animal was returned to its cage to recover from anesthesia.

Protocols

Experiment 1: Effects of PARP inhibition on striatal NAD+ depletion, SIRT1 expression and activity after *in vivo* cerebral oxidative stress. PARP inhibition was induced by intracerebroventricular infusion of 3-aminobenzamide (3AB) as previously described [8]. Briefly, 54 μg of 3AB (A0788, Sigma) was dissolved in 20 μL of 0.9% NaCl (0.2 M). Rats were given 3AB or its vehicle (0.9% NaCl) intracerebroventricularly (i.c.v.) 30 minutes before malonate infusion. This schedule was demonstrated to reduce PAR production and cerebral lesion induced by stroke [21] and OS [8]. Striatum were promptly removed at 4 or 24 hours after malonate for NAD^+ determination, as this latter was depleted at these times (Data S1) and at 6 hours for SIRT1 expression and activity assessment.

Experiment 2: Effects of combination of PARP inhibition with SIRT1 activation or inhibition on neurological deficit and lesion induced by *in vivo* cerebral oxidative stress. For PARP inhibition, 3AB was administrated as in experiment 1. For SIRT1 activation, 0.3 μg of SRT1720 (S1129, Selleckchem), a potent specific activator, was given i.c.v. [22]. The dose chosen was calculated for a final cerebrospinal concentration (1 μM) which permitted to activate SIRT1 *in vitro* [22]. For SIRT1 inhibition, 1 μg of EX527 (6 μM; S1541, Selleckchem) was used and administrated i.c.v. as this schedule was demonstrated to inhibit SIRT1 in the brain [23]. All reagents were dissolved in 0.9% NaCl containing 3.4% of dimethylsulfoxide (DMSO). Rats were given 3AB, SRT1720, EX527, combination of 3AB and SRT1720, combination of 3AB and EX527 or vehicle (3.4% DMSO in 0.9% NaCl) i.c.v. in a volume of 20 μL, 30 minutes before malonate infusion. As the EX527 schedule was demonstrated to inhibit SIRT1 at 6 hours [23] and NAD^+ being decreased between 4 and 24 hours (Data S1), neurological assessments, striatal lesion, SIRT1 expression and activity were performed 6 hours after malonate infusion.

Neurologic Assessment

A neurological assessment was performed in a blind fashion using 3 grading scales:

– the first scale evaluated contralateral sensorimotor function with items whose scores were summed and used as a neurological score [24]; the maximum score was 9 for unoperated rats.
– the second scale was performed to evaluate motor coordination by beam-walking test as previously described [25]; the maximum score was 4 for unoperated rats.
– the third scale measured postural asymmetry of animals using circling test [26]; the maximum score was 2 for unoperated rats.

These 3 scores (sensorimotor, beam-walking and circling) were summed as a global neurological score (GNS) to evaluate the global neurological function. The maximum GNS was 15 for unoperated rats.

Brain Lesion

Brain lesion was assessed by cresyl violet staining of coronal sections (50 μm) [8]. The lesion areas (in mm^2) were measured using a computer image analysis system (Image J, version 1.41) and the distance between respective coronal sections (500 μm) was used to calculate the lesion volume. The brain lesion volume (mm^3) was calculated by integrating the necrotic areas, corrected for edema [27].

NAD^+ Determination

Nicotinamide adenine dinucleotide (NAD^+) was measured on the left striatum as previously described [28,29]. Rats received an overdose of sodium pentobarbital (150 mg/kg, i.p.) and their brains were promptly removed. The left striatum were rapidly dissected on ice, weighed, and immediately homogenized in 2 mL of ice-cold perchlorhydric acid. The homogenates were centri-

fuged for 15 minutes at 1 000 g and 4°C. The supernatant were neutralized (pH 6.8–7.5) with a solution containing 660 mM phosphate buffer pH 7.5: 4 M potassium hydroxide in a 1:1 ratio. Each sample was diluted 1/5 in distilled water. Then, 50 µl of sample were added to 120 µL of the reaction mixture (85 mM bicine, 3.3 mM EDTA, 0.07‰ bovine serum albumin, 0.3 mM 3-[4,5-dimethylthiazol-2-yl]-2,5-diphenyl-tetrazolium bromide, 1.3 mM phenazine ethosulfate, 3% ethanol, 1.7% alcohol dehydrogenase). The colored product formed was then detected at a wavelength of 550 nm with a colorimetric microplate reader (Dynex Technologie). NAD^+ content was expressed in picomoles per milligram of wet tissue (pmol/mg).

Brain Sample Preparation and Protein Determination for Blotting Analyses

Rats received an overdose of sodium pentobarbital (150 mg/kg, i.p.). Their brains were promptly removed, the left striatum isolated, frozen in liquid nitrogen and stored at −40°C.

Nuclear proteins were extracted using a ProteoExtract Subcellular Proteome Extraction Kit (539790, Calbiochem), according to the supplier's instructions, and measured by bicinchoninic acid assay. Samples were diluted in Laemmli buffer (62.5 mM Tris-HCl pH 6.8, 10% glycerol, 5% ß-mercaptoethanol, 4% sodium dodecyl sulfate (SDS), 0.01% bromophenol blue) and heated to 95°C for 10 minutes.

Western-blot of SIRT1

Samples were separated in a 6% SDS-polyacrylamide gel, transferred onto polyvinylidene fluoride (PVDF) membrane (0.45 µm, Millipore) and the free sites on membrane were blocked 20 minutes with a blocking solution (3% non-fat dry milk in tris-buffer saline (TBS; 10 mM Tris-HCl pH 7.6; 142 mM NaCl) and 0.1% Tween 20 (TTBS)). Then, membranes were probed with a rabbit anti-SIRT1 primary antibody (1:1 000; 07–131, Millipore) overnight at 4°C in the blocking solution. After three washing in TTBS, membranes were incubated with donkey anti-rabbit IgG-fluorescein antibody (1:1 000; N1034, GE Healthcare) in the blocking solution for 1 hour at room temperature in obscurity. Then, membranes were incubated with rabbit anti-fluorescein-alkaline phosphatase antibody (1:2 000; A5719, Sigma) following the same schedule. The SIRT1 specific immunoreactivity was visualized using the ECF Western blotting substrate (RPN5785, GE Healthcare), according to the supplier's instructions. Membranes were scanned using Storm 860 apparatus (GE Healthcare) and Image Quant Software (GE Healthcare) was used for quantification. Results were expressed in arbitrary units (AU).

Dot-blot of Acetylated-histone H3

Acetylated-histone H3, assayed by dot-blot, was used to evaluate the nuclear SIRT1 activity [30,31]. Samples (0.5 µg) were quickly added to a PVDF membrane (0.2 µm, Millipore) pre-wet with ethanol. Membranes were washed 5 minutes in methanol and twice in TBS. Unoccupied binding sites were blocked 1h30 with blocking solution containing 3% non-fat dry milk in TBS. Then, the membranes were incubated with a primary antibody, rabbit anti- acetylated-histone H3 (1:6 700; 06-599, Upstate), overnight at 4°C in blocking solution. The acetylated-histone H3 specific immunoreactivity was visualized as described for SIRT1 protein western-blot. Results were expressed in arbitrary units (AU).

Assessment of *in vitro* PARP Activity

Poly(ADP-ribose) (PAR) production was used as a marker of PARP activity [32]. Twenty units of human recombinant PARP (4667-50-EB, Trevigen) was activated by 22 µg of DNA (D4522, Sigma) in a 50 mM tris-HCl buffer pH 8 containing 40 mM $MgCl_2$ and 1 mM dithiotreitol (DTT). Samples were incubated with 1 mg/mL of histone (H5505, Sigma), used as a PAR acceptor, and 11 mM of NAD (L3014, Sigma) during 10 minutes at 25°C. Addition of NAD initiated PARP reaction activity which was evaluated in presence or absence of SIRT1 inhibitor: 0.01 to 20 µM of EX527 (S1541, Selleckchem), and SIRT1 activator: 0.001 to 100 µM of SRT1720 (S1129, Selleckchem). Reaction was stopped by addition of 125 mM tris-HCl buffer pH 6.8 containing 130 mM DTT and 4% SDS, and samples were heated to 95°C for 10 minutes. Samples (1 µL) were quickly added in triplicate to a PVDF membrane (0.45 µm, Millipore) locally pre-wet with ethanol. The membranes were washed 5 minutes twice in bidistilled water and twice in TTBS. Unoccupied binding sites were blocked 1 hour with a blocking solution containing 5% non-fat dry milk in TTBS. Then, the membranes were incubated with a primary antibody, mouse anti-poly-ADP-ribose antibody (1:3 000, 4335-MC-100, Trevigen), overnight at 4°C in blocking solution. Next day, they were washed three times with TTBS and incubated with anti-mouse IgG-peroxydase secondary antibody (1:50 000; A9309, Sigma) for 1 hour at room temperature in the blocking solution. Membranes were washed three times and PAR production was visualized using chemioluminescence ECL Advance Western blotting detection reagent (RPN2135, GE Healthcare). Membranes were exposed onto Kodak Biomax Light film (Z373508, Sigma) which were then scanned using Gel Doc 2000 (Bio-Rad). The PAR expression was quantified using Quantity One software (Bio-Rad). Results were expressed in relative PAR production of control (without test compound; % of control).

Statistical Analysis

Results were expressed as mean ± S.E.M. Figures and analyses were performed using GraphPad Prism 5.0 (GraphPad Software, San Diego, USA). Neurological scores were compared using a Kruskal-Wallis analysis followed by a Mann-Whitney test. Whenever suitable, other results were analysed using one-way ANOVA followed by a Dunnett post-hoc test or two-way ANOVA followed by a Student's t-test. Differences were considered statistically significant when $P<0.05$.

Results

Effect of 3AB on NAD^+ Depletion after *in vivo* Cerebral OS

Non-operated rat had a striatal NAD^+ level of 119±10 pmol/mg of tissue that was not different from that of sham-operated animals receiving 3AB vehicle (105±7 pmol/mg of tissue) (Fig. S1). The NAD^+ level of sham-operated rats receiving 3AB (108±9 pmol/mg of tissue) did not differ from that of sham-operated rats receiving vehicle. Malonate led to a 50% decrease of NAD^+ level at 4 h (66±7 pmol/mg, $P<0.001$) that persisted at 24 h (38±4 pmol/mg, $P<0.001$), demonstrating a NAD^+ depletion. Treatment with 3AB did not modify NAD^+ level at 4 (62±4 pmol/mg of tissue) and 24 h (51±6 pmol/mg of tissue) after malonate.

Effect of 3AB on Nuclear SIRT1 Expression and Activity after *in vivo* Cerebral OS

Non-operated rats had a nuclear SIRT1 expression of 60±12 AU that was not different from that of sham-operated

animals receiving 3AB vehicle (59±3 AU) (Fig. S2A). The nuclear SIRT1 expression of sham-operated rats receiving 3AB (60±6 AU) did not differ from that of sham-operated animals receiving its vehicle. Malonate induced a reduction of SIRT1 expression (43±3 AU, P<0.001) which was exacerbated by 3AB treatment (28±3 AU, P<0.001), showing a worsen SIRT1 decrease when PARP was inhibited during OS.

Non-operated rats had a striatal acetylated-H3 level 27±3 AU (Fig. S2B). There was no difference between sham-operated rats receiving 3AB (30±4 AU) or its vehicle (32±3 AU) and malonate rats receiving 3AB (25±2 AU) or its vehicle (30±3 AU), showing no effect on nuclear SIRT1 activity when PARP was inhibited during OS.

Non-operated rats had a nuclear SIRT1 activity/expression ratio of 0.5±0.1 that was not different from that of sham-operated animals receiving 3AB (0.5±0.1) or its vehicle (0.5±0.1) (Fig. S2C). Malonate did not modify nuclear SIRT1 activity/expression ratio (0.7±0.1), showing no effect of OS on SIRT1 activity. Treatment with 3AB increased nuclear SIRT1 activity/expression ratio (0.9±0.1, P<0.05), showing an increase of nuclear SIRT1 activity relative to its expression when PARP was inhibited during OS.

Effects of 3AB, SRT1720, EX527, 3AB+SRT1720 and 3AB+EX527 Association on Neurological Deficit after *in vivo* Cerebral OS

Non-operated rat had a GNS of 14.4±0.2 (Fig. S3). Malonate decreased the GNS (5.8±0.6, P<0.001), demonstrating a neurological deficit. This deficit was reduced with both 3AB (9.4±0.6, P<0.01) and SRT1720 (11.0±1.0, P<0.01), showing a neurological protection when PARP was inhibited or SIRT1 activated. Treatment with EX527 had no statistically significant effect on the GNS (7.0±1.0), demonstrating that SIRT1 inhibition did not exacerbate the neurological consequence of cerebral OS.

The association of 3AB with SRT1720, the SIRT1 activator, did not modify the neurological recovery (8.6±1.0) induced with 3AB alone (9.4±0.6). The association of 3AB with EX527, the SIRT1 inhibitor, suppressed the neurological improvement (5.6±0.5, P<0.001) induced with 3AB alone, showing a loss of neurological recovery when PARP and SIRT1 were both inhibited.

Effects of 3AB, SRT1720, EX527, 3AB+SRT1720 and 3AB+EX527 Association on Striatal Lesion after *in vivo* Cerebral OS

Malonate induced a striatal lesion volume of 46±1 mm^3 (Fig. S4). This lesion was reduced with both 3AB (30±5 mm^3, P<0.01) and SRT1720 (32±4 mm^3, P<0.05), showing histological protection when PARP was inhibited or SIRT1 activated. Treatment with EX527 had no effect on the striatal lesion volume (40±6 mm^3), demonstrating that SIRT1 inhibition did not exacerbate the histological consequence of *in vivo* cerebral OS.

The association of 3AB with SRT1720, the SIRT1 activator, did not modify the reduction of brain lesion (37±4 mm^3) induced with 3AB alone. These results showed no additive effect of PARP inhibition and SIRT1 activation. The association of 3AB with EX527, the SIRT1 inhibitor, suppressed the lesion reduction (47±3 mm^3; P<0.01) induced with 3AB alone, showing a loss of neuroprotective effect when PARP and SIRT1 were both inhibited.

Dose-effect of SRT1720 and EX527 on PARP Activity *in vitro*

SRT1720 at the dose of 100 μM reduced about approximately 25% PAR production (73%±11%) whereas the lower doses had no effect (0.01 μM: 96%±17%; 0.1 μM: 87%±19%; 1 μM: 89%±16%; 5 μM: 81%±18%; 10 μM: 92%±16%) (Fig. S5A). These results demonstrated a PARP inhibitory effect of SRT1720 at high dose.

EX527 had no effect on PAR production by PARP, whatever the dose tested (0.01 μM: 88%±5%; 0.1 μM: 85%±9%; 0.4 μM: 90%±10%; 1.6 μM: 104%±9%; 6 μM: 87%±5%; 20 μM: 102%±5%) (Fig. S5B).

Therefore, both doses of SRT1720 (0.3 μg corresponding to a calculated cerebrospinal concentration of 1 μM) and EX527 (1 μg corresponding to a calculated cerebrospinal concentration of 6 μM) tested *in vivo* had no effect on PARP activity *in vitro*.

Discussion

PARP activation is a major cause of neuronal death in acute cerebrovascular diseases like stroke and trauma, where OS is important [6]. Firstly, energetic failure was suggested to explain, at least in part, the deleterious effects of PARP activation [33,34]. The present study showed that *in vivo* cerebral OS induced a NAD^+ decrease *in vivo* and that 3AB, the PARP inhibitor, did not prevent it. However, 3AB increased SIRT1 activity after *in vivo* cerebral OS. SIRT1 activator, SRT1720, promoted neurological recovery and striatal lesion reduction, whereas SIRT1 inhibitor, EX527, did not worsen neurological and histological consequences of OS suggesting (1) the absence of endogenous SIRT1 activation during cerebral OS, (2) nuclear SIRT1 activation when PARP is inhibited by 3AB during cerebral OS, and (3) beneficial effect of exogenous SIRT1 activation by SRT1720 during cerebral OS. Moreover, the SIRT1 inhibitor, EX527, suppressed the beneficial effects of PARP inhibition on neurological deficit and lesion consecutive to OS. These data suggested that SIRT1 activation was implicated in beneficial effects of PARP inhibition in cerebral *in vivo* OS (Fig. S6).

In vivo cerebral OS induced a 40% decrease at 4 hours and a 65% decrease at 24 hours in striatal level of NAD^+. These results were in accordance with studies showing NAD^+ loss of 30% 2 hours after traumatic brain injury [35] and 30 to 80% in the first 24 hours following experimental stroke [36,37]. *In vitro* data showed that PARP inhibition restored NAD^+ level and decreased cell death induced with hydrogen peroxide [9,13–15]. Same results were obtained with peroxynitrite production or DNA breaks in cortical neurons culture [34]. *In vivo*, 3AB, the PARP inhibitor, was shown to reduce NAD^+ loss and infarction consecutive to cerebral ischemia [21]. Although 3AB inhibited PARP activation and reduced striatal lesion in the cerebral OS model [8], this dose failed to restore NAD^+ level observed at 4 and 24 hours following *in vivo* cerebral OS. Surprisingly, our data suggested that NAD^+ depletion is not due to PARP activation and may originate from other NAD^+-dependent enzymes such as SIRT1. However, it has been shown that SIRT1 activity was decreased following *in vitro* OS [9,14,15] and PARP inhibition allowed to restore its activity [9,15]. *In vivo*, both SIRT1 expression and activity were reduced 6 hours following experimental cerebral ischemia [37] and its expression was decreased 7 and 14 days after traumatic brain injury [38]. In human, a *post-mortem* study showed a SIRT1 expression decrease in brain of Alzheimer disease patients, a chronic neurodegenerative pathology where OS is also present [39]. Interestingly, SIRT1 expression and activity were decreased during experimental heart failure, and PARP inhibition

with 3AB induced restoration of SIRT1 expression [13]. To date, there was still no information about the effects of PARP inhibition on SIRT1 in experimental models of stroke and TBI. Our present study demonstrated that 3AB increased nuclear SIRT1 activity/expression ratio demonstrating that inhibition of PARP could promote endogenous SIRT1 activation after *in vivo* cerebral OS. The benefit of PARP inhibition, with 3AB using the same protocol, was already showed on striatal lesion during cerebral OS by our group [8], and was associated with a reduced neurological deficit consecutive to cerebral OS. As 3AB did not directly activate SIRT1 [11], its neuroprotective effects may then originate from SIRT1 activity increase induced by PARP inhibition following *in vivo* cerebral OS.

Then, we assessed the implication of SIRT1 activity in the beneficial effects of 3AB on neurological deficit and striatal lesion induced by *in vivo* OS using SRT1720, a SIRT1 activator [22], and EX527, a SIRT1 inhibitor [23]. As there was no data in the literature, we first studied *in vitro* dose-response of SRT1720 and EX527 on PARP activity in order to ensure the absence of interaction with the enzyme activity that could potentially explain *in vivo* effects. In our experimental *in vitro* conditions, PARP was brought to optimal activity conditions [32] with, or not, SIRT1 activator or inhibitor. The production of PAR by PARP was blocked with both PARP inhibitors, 3AB and PJ34 (unpublished data). SRT1720 had no effect on PARP activity at the lower doses but the high dose, 100 µM, decreased PARP activity of 25%, whereas EX527 was devoid of any effect. This study is the first one showing the ability of SRT1720 to inhibit, partially, PARP activity. Therefore, both doses of SRT1720 (0.3 µg corresponding to a calculated cerebrospinal concentration of 1 µM) and EX527 (1 µg corresponding to a calculated cerebrospinal concentration of 6 µM) tested *in vivo* had no effect on PARP activity *in vitro*.

The benefit of SIRT1 activation with SRT1720 was described *in vivo* in metabolic diseases [22,40–44], leading to clinical trials of similar molecules [45]. However, there was no data on its effects in cerebral *in vivo* OS and cerebrovascular diseases. As there was no information on the passage of SRT1720 across the blood-brain barrier, it was directly administrated into the cerebrospinal fluid at a dose calculated from *in vitro* data showing 4 time SIRT1 activation [22]. For the first time, SRT1720 was demonstrated to reduce both neurological deficit and striatal lesion consecutive to OS, suggesting that SIRT1 activation is beneficial on neurological and histological consequences of an *in vivo* cerebral OS. Previous study had shown that resveratrol, a SIRT activator [46], protects neurons from cell death following cardiac arrest in a SIRT-dependent way [47]. Neuroprotective effects of resveratrol are known [48–50] but are often attributed to its antioxidant and anti-inflammatory properties [51]. SIRT1 activation alone could repress PARP activity. Indeed Kolthur-Seetharam and colleagues [52] had showed that SIRT1 deletion increased PARP activity *in vitro* under OS conditions. Moreover, SIRT1-mediated deacetylation of PARP inhibits its enzymatic activity [53]. 3AB+SRT1720 association had no more beneficial effects on the consequences of *in vivo* cerebral OS than each strategy given alone. Our result suggested activation of SIRT1, with SRT1720, may repress PARP activity thus explaining the absence of additional effect of the 3AB+SRT1720 association.

EX527, a potent SIRT1 inhibitor [54] that has been demonstrated to inhibit SIRT1 6 hours after its i.c.v. administration [23], modified neither functional nor histological consequences of OS, supporting that SIRT1 was not activated while PARP was activated in cerebral OS. In addition, we showed that 3AB+EX527 association suppressed both the neurological improvement and the reduction of striatal lesion induced by 3AB alone. To our knowledge, these data demonstrated, for the first time, that SIRT1 activation plays a key role in the neuroprotective effects of PARP inhibition during an *in vivo* cerebral OS (Fig. S6).

Beneficial effects of PARP inhibition are explained, partly, by the major role of PARP in promoting inflammation [2,55]. Indeed PARP regulates inflammation as it acts also as a direct co-activator of the transcription factor nuclear factor-kappa B (NF-κB) resulting in the synthesis of pro-inflammatory mediators [56]. At the same time, very recent studies have demonstrated that SIRT1 exhibited also anti-inflammatory properties [57] through promotion of deacetylation of p65 subunit of NF-κB [58,59]. SRT1720 has been shown to suppress inflammatory cell infiltration and cytokine production in a mouse model of asthma [60]. Thus PARP inhibition may promote directly a decrease of PARP interaction with NF-κB and/or indirectly an increase of SIRT1 activity inducing a deacetylation of p65 subunit of NF-κB, leading to the well-described anti-inflammatory effects of PARP inhibitors. The wide range of future studies evaluating the involvement of SIRT1 in the various effects of PARP inhibition promises close relationship between PARP and SIRT1.

Supporting Information

Figure S1 3-aminobenzamide did not prevent NAD^+ depletion consecutive to cerebral oxidative stress. Effects of 3-aminobenzamide (3AB) on NAD^+ depletion 4 and 24 hours after *in vivo* cerebral oxidative stress (n = 7–9). Data are presented as mean ± S.E.M. Differences were evaluated by two-way ANOVA followed by Student t-test group comparisons. ***P< 0.001.
(TIFF)

Figure S2 3-aminobenzamide decreased nuclear SIRT1 expression without modifying its activity, demonstrating basal SIRT1 activity increase. Effects of 3-aminobenzamide (3AB) on (A) nuclear SIRT1 expression, (B) acetylated-histone H3 expression and (C) nuclear SIRT1 activity/expression ratio 6 hours after *in vivo* cerebral oxidative stress (n = 5–9). Data are presented as mean ± S.E.M. Differences were evaluated by two-way ANOVA followed by Student t-test group comparisons. **P<0.01.
(TIFF)

Figure S3 Both 3-aminobenzamide and SRT1720 reduced neurological deficit whereas EX527 blocked beneficial effects of PARP inhibition. Effects of 3AB, SRT1720, EX527, 3AB+SRT1720 and 3AB+EX527 association on neurological deficit 6 hours after *in vivo* cerebral oxidative stress (n = 7–11). Data are presented as mean ± S.E.M. Differences were evaluated Kruskal-Wallis analysis followed by Mann-Whitney test group comparisons. **P<0.01, ***P<0.001.
(TIFF)

Figure S4 Both 3-aminobenzamide and SRT1720 reduced striatal lesion whereas EX527 blocked beneficial effects of PARP inhibition. Effects of 3AB, SRT1720, EX527, 3AB+SRT1720 and 3AB+EX527 association on striatal lesion 6 hours after *in vivo* cerebral oxidative stress (n = 7–11). (A) Representative photographs. (B) Representative histograms. Data are presented as mean ± S.E.M. Differences were evaluated by two-way ANOVA followed by Student t-test group comparisons. *P<0.05, **P<0.01.
(TIFF)

Figure S5 Dose-effect of SRT1720 and EX527 on PARP activity *in vitro*. Dose-effect of (A) SRT1720, a SIRT1 activator and (B) EX527, a SIRT1 inhibitor, on relative PAR production.

Data are expressed in % of control as mean ± S.E.M. Differences were evaluated by one way-ANOVA followed by Dunnett test. *P<0.05 and **P<0.01.
(TIFF)

Figure S6 Schematic interrelationship between PARP, SIRT1 and neuroprotection during *in vivo* cerebral oxidative stress. (A) in physiological conditions, PARP and SIRT1 are both NAD^+-dependent enzymes whose activities are probably in balance; (B) in oxidative stress conditions, PARP is hyperactivated causing NAD^+ decrease. (C) in oxidative stress conditions, EX527, a SIRT1 inhibitor, is not deleterious suggesting that an endogenous SIRT1 activation is not present when PARP is hyperactivated and NAD^+ is consumed (B). (D) in oxidative stress conditions, SRT1720, a SIRT1 activator, is neuroprotective suggesting beneficial effects of an exogenous SIRT1 activation. (E) in oxidative stress conditions, PARP hyperactivation contributes to neurological deficit and striatal lesion showing a deleterious role of PARP. Its inhibition increases nuclear SIRT1 activity that may, partly, explain the neuroprotective effects of 3AB. (F) in oxidative stress conditions, neuroprotective effects of 3AB are suppressed by EX527, suggesting that SIRT1 activation is implicated in neuroprotective effect of PARP inhibition during *in vivo* cerebral oxidative stress.
(TIFF)

Data S1 Time course of NAD^+ level after *in vivo* cerebral oxidative stress (n = 7/group). Data are expressed as mean ± S.E.M. Differences were evaluated by one way-ANOVA followed by Dunnett test. **P<0.01 and ***P<0.001.
(TIFF)

Acknowledgments

We would like to thank Dr Sylvain Broussy for study of SRT1720 stability, Dr Emmanuel Curis for validation of statistical analyses and Pr Isabelle Margaill for critical reading of the manuscript.

Author Contributions

Conceived and designed the experiments: CG VB BP. Performed the experiments: CG BP. Analyzed the data: CG VB. Contributed reagents/materials/analysis tools: BP CML MP VB. Wrote the paper: CG VB CML BP MP.

References

1. Margaill I, Plotkine M, Lerouet D (2005) Antioxidant strategies in the treatment of stroke. Free Radic Biol Med 39: 429–443. doi:10.1016/j.freeradbiomed.2005.05.003.
2. Besson VC (2009) Drug targets for traumatic brain injury from poly(ADP-ribose)polymerase pathway modulation. Br J Pharmacol 157: 695–704. doi:10.1111/j.1476-5381.2009.00229.x.
3. Hall ED, Vaishnav RA, Mustafa AG (2010) Antioxidant therapies for traumatic brain injury. Neurotherapeutics 7: 51–61. doi:10.1016/j.nurt.2009.10.021.
4. Abeti R, Abramov AY, Duchen MR (2011) Beta-amyloid activates PARP causing astrocytic metabolic failure and neuronal death. Brain 134 (Pt 6): 1658–1672. doi: 10.1093/brain/awr104.
5. Strosznajder RP, Czubowicz K, Jesko H, Strosznajder JB (2010) Poly(ADP-ribose) metabolism in brain and its role in ischemia pathology. Mol Neurobiol 41: 187–196. doi:10.1007/s12035-010-8124-6.
6. Komjáti K, Besson VC, Szabó C (2005) Poly (adp-ribose) polymerase inhibitors as potential therapeutic agents in stroke and neurotrauma. Curr Drug Targets CNS Neurol Disord 4: 179–194.
7. Schraufstatter IU, Hyslop PA, Hinshaw DB, Spragg RG, Sklar LA, et al. (1986) Hydrogen peroxide-induced injury of cells and its prevention by inhibitors of poly(ADP-ribose) polymerase. Proc Natl Acad Sci U S A 83: 4908–4912.
8. Besson VC, Margaill I, Plotkine M, Marchand-Verrecchia C (2003) Deleterious activation of poly(ADP-ribose)polymerase-1 in brain after in vivo oxidative stress. Free Radic Res 37: 1201–1208.
9. Bai P, Cantó C, Oudart H, Brunyánszki A, Cen Y, et al. (2011) PARP-1 inhibition increases mitochondrial metabolism through SIRT1 activation. Cell Metab 13: 461–468. doi:10.1016/j.cmet.2011.03.004.
10. Michan S, Sinclair D (2007) Sirtuins in mammals: insights into their biological function. Biochem J 404: 1–13. doi:10.1042/BJ20070140.
11. Luo J, Nikolaev AY, Imai S, Chen D, Su F, et al. (2001) Negative control of p53 by Sir2alpha promotes cell survival under stress. Cell 107: 137–148.
12. Araki T, Sasaki Y, Milbrandt J (2004) Increased nuclear NAD biosynthesis and SIRT1 activation prevent axonal degeneration. Science 305: 1010–1013. doi:10.1126/science.1098014.
13. Pillai JB, Isbatan A, Imai S, Gupta MP (2005) Poly(ADP-ribose) polymerase-1-dependent cardiac myocyte cell death during heart failure is mediated by NAD+ depletion and reduced Sir2alpha deacetylase activity. J Biol Chem 280: 43121–43130. doi:10.1074/jbc.M506162200.
14. Furukawa A, Tada-Oikawa S, Kawanishi S, Oikawa S (2007) H2O2 accelerates cellular senescence by accumulation of acetylated p53 via decrease in the function of SIRT1 by NAD+ depletion. Cell Physiol Biochem 20: 45–54. doi:10.1159/000104152.
15. Caito S, Hwang J-W, Chung S, Yao H, Sundar IK, et al. (2010) PARP-1 inhibition does not restore oxidant-mediated reduction in SIRT1 activity. Biochem Biophys Res Commun 392: 264–270. doi:10.1016/j.bbrc.2009.12.161.
16. Ying W, Alano CC, Garnier P, Swanson RA (2005) NAD+ as a metabolic link between DNA damage and cell death. J Neurosci Res 79: 216–223. doi:10.1002/jnr.20289.
17. Paucard A, Besson VC, Plotkine M, Margaill I (2005) Time course of oxidative stress, lesion and edema after intrastriatal injection of malonate in rat: effect of alpha-phenyl-N-tert-butylnitrone. Fundam Clin Pharmacol 19: 57–64. doi:10.1111/j.1472-8206.2004.00297.x.
18. Schulz JB, Henshaw DR, Siwek D, Jenkins BG, Ferrante RJ, et al. (1995) Involvement of free radicals in excitotoxicity in vivo. J Neurochem 64: 2239–2247.
19. Paxinos G, Watson C (1986) The Rat Brain in Stereotaxic Coordinates. Academic Press.
20. Paxinos G, Watson C (2007) The Rat Brain in Stereotaxic Coordinates. Academic Press. 451 p.
21. Endres M, Wang ZQ, Namura S, Waeber C, Moskowitz MA (1997) Ischemic brain injury is mediated by the activation of poly(ADP-ribose)polymerase. J Cereb Blood Flow Metab 17: 1143–1151. doi:10.1097/00004647-199711000-00002.
22. Milne JC, Lambert PD, Schenk S, Carney DP, Smith JJ, et al. (2007) Small molecule activators of SIRT1 as therapeutics for the treatment of type 2 diabetes. Nature 450: 712–716. doi:10.1038/nature06261.
23. Velásquez DA, Martínez G, Romero A, Vázquez MJ, Boit KD, et al. (2011) The central Sirtuin 1/p53 pathway is essential for the orexigenic action of ghrelin. Diabetes 60: 1177–1185. doi:10.2337/db10-0802.
24. Besson VC, Croci N, Boulu RG, Plotkine M, Marchand-Verrecchia C (2003) Deleterious poly(ADP-ribose)polymerase-1 pathway activation in traumatic brain injury in rat. Brain Res 989: 58–66.
25. Chen XR, Besson VC, Beziaud T, Plotkine M, Marchand-Leroux C (2008) Combination therapy with fenofibrate, a peroxisome proliferator-activated receptor alpha agonist, and simvastatin, a 3-hydroxy-3-methylglutaryl-coenzyme A reductase inhibitor, on experimental traumatic brain injury. J Pharmacol Exp Ther 326: 966–974. doi:10.1124/jpet.108.140368.
26. Bederson JB, Pitts LH, Tsuji M, Nishimura MC, Davis RL, et al. (1986) Rat middle cerebral artery occlusion: evaluation of the model and development of a neurologic examination. Stroke 17: 472–476.
27. Golanov EV, Reis DJ (1995) Contribution of cerebral edema to the neuronal salvage elicited by stimulation of cerebellar fastigial nucleus after occlusion of the middle cerebral artery in rat. J Cereb Blood Flow Metab 15: 172–174. doi:10.1038/jcbfm.1995.19.
28. Bernofsky C, Swan M (1973) An improved cycling assay for nicotinamide adenine dinucleotide. Anal Biochem 53: 452–458.
29. Shah GM, Poirier D, Duchaine C, Brochu G, Desnoyers S, et al. (1995) Methods for biochemical study of poly(ADP-ribose) metabolism in vitro and in vivo. Anal Biochem 227: 1–13. doi:10.1006/abio.1995.1245.
30. Tanno M, Sakamoto J, Miura T, Shimamoto K, Horio Y (2007) Nucleocytoplasmic shuttling of the NAD+-dependent histone deacetylase SIRT1. J Biol Chem 282: 6823–6832. doi:10.1074/jbc.M609554200.
31. Shinmura K, Tamaki K, Bolli R (2008) Impact of 6-mo caloric restriction on myocardial ischemic tolerance: possible involvement of nitric oxide-dependent increase in nuclear Sirt1. Am J Physiol Heart Circ Physiol 295: H2348–2355. doi:10.1152/ajpheart.00602.2008.
32. Cosi C, Cavalieri E, Carcereri de Prati A, Marien M, Suzuki H (2000) Effects of kainic acid lesioning on poly(ADP-ribose) polymerase (PARP) activity in the rat striatum in vivo. Amino Acids 19: 229–237.
33. Ying W (2008) NAD+/NADH and NADP+/NADPH in cellular functions and cell death: regulation and biological consequences. Antioxid Redox Signal 10: 179–206. doi:10.1089/ars.2007.1672.
34. Alano CC, Garnier P, Ying W, Higashi Y, Kauppinen TM, et al. (2010) NAD+ depletion is necessary and sufficient for poly(ADP-ribose) polymerase-1-

mediated neuronal death. J Neurosci 30: 2967–2978. doi:10.1523/JNEUROSCI.5552-09.2010.

35. Clark RSB, Vagni VA, Nathaniel PD, Jenkins LW, Dixon CE, et al. (2007) Local administration of the poly(ADP-ribose) polymerase inhibitor INO-1001 prevents NAD+ depletion and improves water maze performance after traumatic brain injury in mice. J Neurotrauma 24: 1399–1405. doi:10.1089/neu.2007.0305.
36. Yang J, Klaidman LK, Nalbandian A, Oliver J, Chang ML, et al. (2002) The effects of nicotinamide on energy metabolism following transient focal cerebral ischemia in Wistar rats. Neurosci Lett 333: 91–94.
37. Liu D, Gharavi R, Pitta M, Gleichmann M, Mattson MP (2009) Nicotinamide prevents NAD+ depletion and protects neurons against excitotoxicity and cerebral ischemia: NAD+ consumption by SIRT1 may endanger energetically compromised neurons. Neuromolecular Med 11: 28–42. doi:10.1007/s12017-009-8058-1.
38. Wu A, Ying Z, Gomez-Pinilla F (2007) Omega-3 fatty acids supplementation restores mechanisms that maintain brain homeostasis in traumatic brain injury. J Neurotrauma 24: 1587–1595. doi:10.1089/neu.2007.0313.
39. Julien C, Tremblay C, Emond V, Lebbadi M, Salem N Jr, et al. (2009) Sirtuin 1 reduction parallels the accumulation of tau in Alzheimer disease. J Neuropathol Exp Neurol 68: 48–58. doi:10.1097/NEN.0b013e3181922348.
40. Feige JN, Lagouge M, Canto C, Strehle A, Houten SM, et al. (2008) Specific SIRT1 activation mimics low energy levels and protects against diet-induced metabolic disorders by enhancing fat oxidation. Cell Metab 8: 347–358. doi:10.1016/j.cmet.2008.08.017.
41. Yamazaki Y, Usui I, Kanatani Y, Matsuya Y, Tsuneyama K, et al. (2009) Treatment with SRT1720, a SIRT1 activator, ameliorates fatty liver with reduced expression of lipogenic enzymes in MSG mice. Am J Physiol Endocrinol Metab 297: E1179–1186. doi:10.1152/ajpendo.90997.2008.
42. Yoshizaki T, Milne JC, Imamura T, Schenk S, Sonoda N, et al. (2009) SIRT1 exerts anti-inflammatory effects and improves insulin sensitivity in adipocytes. Mol Cell Biol 29: 1363–1374. doi:10.1128/MCB.00705-08.
43. Yoshizaki T, Schenk S, Imamura T, Babendure JL, Sonoda N, et al. (2010) SIRT1 inhibits inflammatory pathways in macrophages and modulates insulin sensitivity. Am J Physiol Endocrinol Metab 298: E419–428. doi:10.1152/ajpendo.00417.2009.
44. Minor RK, Baur JA, Gomes AP, Ward TM, Csiszar A, et al. (2011) SRT1720 improves survival and healthspan of obese mice. Sci Rep 1: 70. doi:10.1038/srep00070.
45. Hoffmann E, Wald J, Lavu S, Roberts J, Beaumont C, et al. (2012) Pharmacokinetics and Tolerability of SRT2104, a First-In-Class Small Molecule Activator of SIRT1, after Single and Repeated Oral Administration in Man. British journal of clinical pharmacology. doi:10.1111/j.1365-2125.2012.04340.x.
46. Howitz KT, Bitterman KJ, Cohen HY, Lamming DW, Lavu S, et al. (2003) Small molecule activators of sirtuins extend Saccharomyces cerevisiae lifespan. Nature 425: 191–196. doi:10.1038/nature01960.
47. Della-Morte D, Dave KR, DeFazio RA, Bao YC, Raval AP, et al. (2009) Resveratrol pretreatment protects rat brain from cerebral ischemic damage via a sirtuin 1-uncoupling protein 2 pathway. Neuroscience 159: 993–1002. doi:10.1016/j.neuroscience.2009.01.017.
48. Gupta YK, Chaudhary G, Sinha K, Srivastava AK (2001) Protective effect of resveratrol against intracortical FeCl3-induced model of posttraumatic seizures in rats. Methods Find Exp Clin Pharmacol 23: 241–244.
49. Sinha K, Chaudhary G, Gupta YK (2002) Protective effect of resveratrol against oxidative stress in middle cerebral artery occlusion model of stroke in rats. Life Sci 71: 655–665.
50. Wang Q, Xu J, Rottinghaus GE, Simonyi A, Lubahn D, et al. (2002) Resveratrol protects against global cerebral ischemic injury in gerbils. Brain Res 958: 439–447.
51. Zhang F, Liu J, Shi J-S (2010) Anti-inflammatory activities of resveratrol in the brain: role of resveratrol in microglial activation. Eur J Pharmacol 636: 1–7. doi:10.1016/j.ejphar.2010.03.043.
52. Kolthur-Seetharam U, Dantzer F, McBurney MW, De Murcia G, Sassone-Corsi P (2006) Control of AIF-mediated cell death by the functional interplay of SIRT1 and PARP-1 in response to DNA damage. Cell Cycle 5: 873–877.
53. Rajamohan SB, Pillai VB, Gupta M, Sundaresan NR, Birukov KG, et al. (2009) SIRT1 promotes cell survival under stress by deacetylation-dependent deactivation of poly(ADP-ribose) polymerase 1. Mol Cell Biol 2009 29(15): 4116–29. doi: 10.1128/MCB.00121-09.
54. Solomon JM, Pasupuleti R, Xu L, McDonagh T, Curtis R, et al. (2006) Inhibition of SIRT1 catalytic activity increases p53 acetylation but does not alter cell survival following DNA damage. Mol Cell Biol 26: 28–38. doi:10.1128/MCB.26.1.28-38.2006.
55. Ba X, Garg NJ (2011) Signaling mechanism of poly(ADP-ribose) polymerase-1 (PARP-1) in inflammatory diseases. Am J Pathol 178: 946–955. doi:10.1016/j.ajpath.2010.12.004.
56. Oliver FJ, Ménissier-de Murcia J, Nacci C, Decker P, Andriantsitohaina R, et al. (1999) Resistance to endotoxic shock as a consequence of defective NF-kappaB activation in poly (ADP-ribose) polymerase-1 deficient mice. EMBO J 18: 4446–4454. doi:10.1093/emboj/18.16.4446.
57. Xie J, Zhang X, Zhang L (2013) Negative regulation of inflammation by SIRT1. Pharmacological Research 67: 60–67. doi:10.1016/j.phrs.2012.10.010.
58. Yang H, Zhang W, Pan H, Feldser HG, Lainez E, et al. (2012) SIRT1 activators suppress inflammatory responses through promotion of p65 deacetylation and inhibition of NF-κB activity. PLoS ONE 7: e46364. doi:10.1371/journal.pone.0046364.
59. Kauppinen TM, Gan L, Swanson RA (2013) Poly(ADP-ribose) polymerase-1-induced NAD(+) depletion promotes nuclear factor-κB transcriptional activity by preventing p65 de-acetylation. Biochim Biophys Acta 1833: 1985–1991. doi:10.1016/j.bbamcr.2013.04.005.
60. Ichikawa T, Hayashi R, Suzuki K, Imanishi S, Kambara K, et al. (2013) Sirtuin 1 activator SRT1720 suppresses inflammation in an ovalbumin-induced mouse model of asthma. Respirology 18: 332–339. doi:10.1111/j.1440-1843.2012.02284.x.
